# Achieving the dual goals of biomass production and soil rehabilitation with sown pasture on marginal cropland: Evidence from a multi-year field experiment in Northeast Inner Mongolia

**DOI:** 10.3389/fpls.2022.985864

**Published:** 2022-09-29

**Authors:** Lijun Xu, Da Li, Di Wang, Liming Ye, Yingying Nie, Huajun Fang, Wei Xue, Chunli Bai, Eric Van Ranst

**Affiliations:** ^1^ Institute of Agricultural Resources and Regional Planning, Chinese Academy of Agricultural Sciences, Beijing, China; ^2^ Department of Grassland Research, Baicheng Institute of Animal Husbandry, Baicheng, China; ^3^ Department of Geology, Ghent University, Ghent, Belgium; ^4^ Institute of Geographic Sciences and Natural Resources Research, Chinese Academy of Sciences, Beijing, China; ^5^ Grassland Research Institute, Inner Mongolia Academy of Agricultural and Animal Husbandry Sciences, Hohhot, China

**Keywords:** soil quality, land restoration, grazing pressure, alfalfa, smooth bromegrass, cropland-grassland system, Medicago sativa L, Bromus inermis L.

## Abstract

Grassland is the primary land use in China but has experienced severe degradation in recent decades due to overgrazing and conversion to agricultural production. Here, we conducted a field experiment in northeastern Inner Mongolia to test the effectiveness of sown pastures in lowering the grazing pressure on grasslands and raising the quality of marginal soils. Alfalfa and smooth bromegrass monocultures and mixture were sown in a marginal cropland field in Hulunber in June 2016. Biomass productivity, soil physicochemical, and biological properties were monitored annually from 2016 to 2020. The results showed that the marginal cropland soil responded consistently positively to sown pastures for major soil properties. Soil organic carbon (SOC) and total nitrogen (TN) increased by 48 and 21%, respectively, from 2016 to 2020 over the 0-60 cm soil depth range. Soil microbes responded proactively too. The soil microbial biomass C (SMBC) and N (SMBN) increased by 117 and 39%, respectively, during the period of 2016-2020. However, by the end of the experiment, the soil of a natural grassland field, which was included in the experiment as a control, led the sown pasture soil by 28% for SOC, 35% for TN, 66% for SMBC, and 96% for SMBN. Nevertheless, the natural grassland soil’s productive capacity was inferior to that of the sown pasture soil. The average aboveground biomass productivity of sown pastures was measured at 8.4 Mg ha^-1^ in 2020, compared to 5.0 Mg ha^-1^ for natural grassland, while the root biomass of sown pastures was averaged at 7.5 Mg ha^-1^, leading the natural grassland by 15%. Our analyses also showed that the sown pastures’ biomass productivity advantage had a much-neglected potential in natural grassland protection. If 50% of the available marginal cropland resources in Hulunber under the current environmental protection law were used for sown pastures, the livestock grazing pressure on the natural grasslands would decrease by a big margin of 38%. Overall, these results represent systematic empirical and analytical evidence of marginal cropland soil’s positive responses to sown pastures, which shows clearly that sown pasture is a valid measure both for soil rehabilitation and biomass production.

## 1 Introduction

Grassland covers about 40% of the Earth’s surface and 69% of the global agricultural land area (Bardgett et al., 2021). One-third of the global terrestrial carbon (C) stock is stored in the grassland ecosystem, where majority of this C resides in the soils (Guo and Gifford, 2002). Grassland is also one of the most critical ecosystems that provide extensive services ranging from soil and water conservation to food provisioning, and further to climate stabilization (Mbaabu et al., 2020). However, the temperate meadow steppe in northern China – the dominant form of grassland in eastern Eurasia – had experienced severe degradation in recent decades as a result of development-induced overgrazing and large-scale conversion into intensive agricultural production (Deng et al., 2014; Li et al., 2018). Overall, 59% of the soil organic C stock has been lost through the conversion of grassland to cropland (Guo and Gifford, 2002). It is in this context that sown pasture has been put forward as an option to cope with the adverse effects of grassland-cropland conversion (Xu et al., 2020). On the one hand, sown pasture in scale provides an opportunity to raise the forage production by a factor of 10-20, thus effectively relaxing the bottleneck limit of forage supply on livestock rearing in captivity, which represents a plausible pathway to lower the grazing intensity on the natural grasslands (Wu et al., 2010; Bardgett et al., 2021). On the other hand, a decreased stocking rate in China’s largely overgrazed grassland will provide an opportunity to build a more balanced and resilient plant-soil system (Dubeux and Sollenberger, 2020).

Despite the potential benefits of sown pastures in the fields of combating grassland degradation, improving food security and consolidating climate mitigation (Wu et al., 2010; O’Mara, 2012; Zhang et al., 2015; Wells et al., 2022), systematic evidence showing the plant-soil system’s response to sown pasture is still lacking. Moreover, apparent differences exist between the soil properties of the grassland ecosystem and those of the cropland ecosystem (Qi et al., 2012; Zhang et al., 2018), meaning that expertise obtained from the past efforts of sown pastures on degraded natural grasslands (Li et al., 2018) may *not* apply well to similar settings on, e.g., abandoned croplands. The current environmental protection and ecological restoration policies in China encourage the conversion of marginal croplands to forest- and grasslands with economic compensations (Deng et al., 2014; Delang and Yuan, 2015), especially in traditionally non-farming regions such as Inner Mongolia. This represents an opportunity to utilize the set-aside marginal cropland resources in the agropastoral ecotone in northern China for sown pasture, using locally suitable forage species such as alfalfa (Xu et al., 2021) and smooth bromegrass (Malhi et al., 2002). In this paper, we aim to provide quantitative evidence in how a marginal cropland soil would respond to the establishment of perennial plant species by conducting a multi-year field experiment in an Inner Mongolia meadow steppe region. More specifically, the objectives of this paper are (1) to quantitatively determine soil responses to sown pastures of alfalfa and smooth bromegrass on abandoned cropland in terms of physicochemical and (micro)biological properties during a five-year period; (2) to provide direct year-by-year comparisons of biomass production with a permanent grassland that was included as part of the experiment; (3) to elaborate sown pasture’s multiple benefits including increased soil carbon sequestration, improved soil quality, higher forage production, and lower grazing pressure on the natural grassland; and (4) to derive implications for land conservation and formulate policy recommendations for maximizing the socioeconomic and the ecological values of sown pasture in northern China.

## 2 Materials and methods

### 2.1 Study site

**Figure 1:**
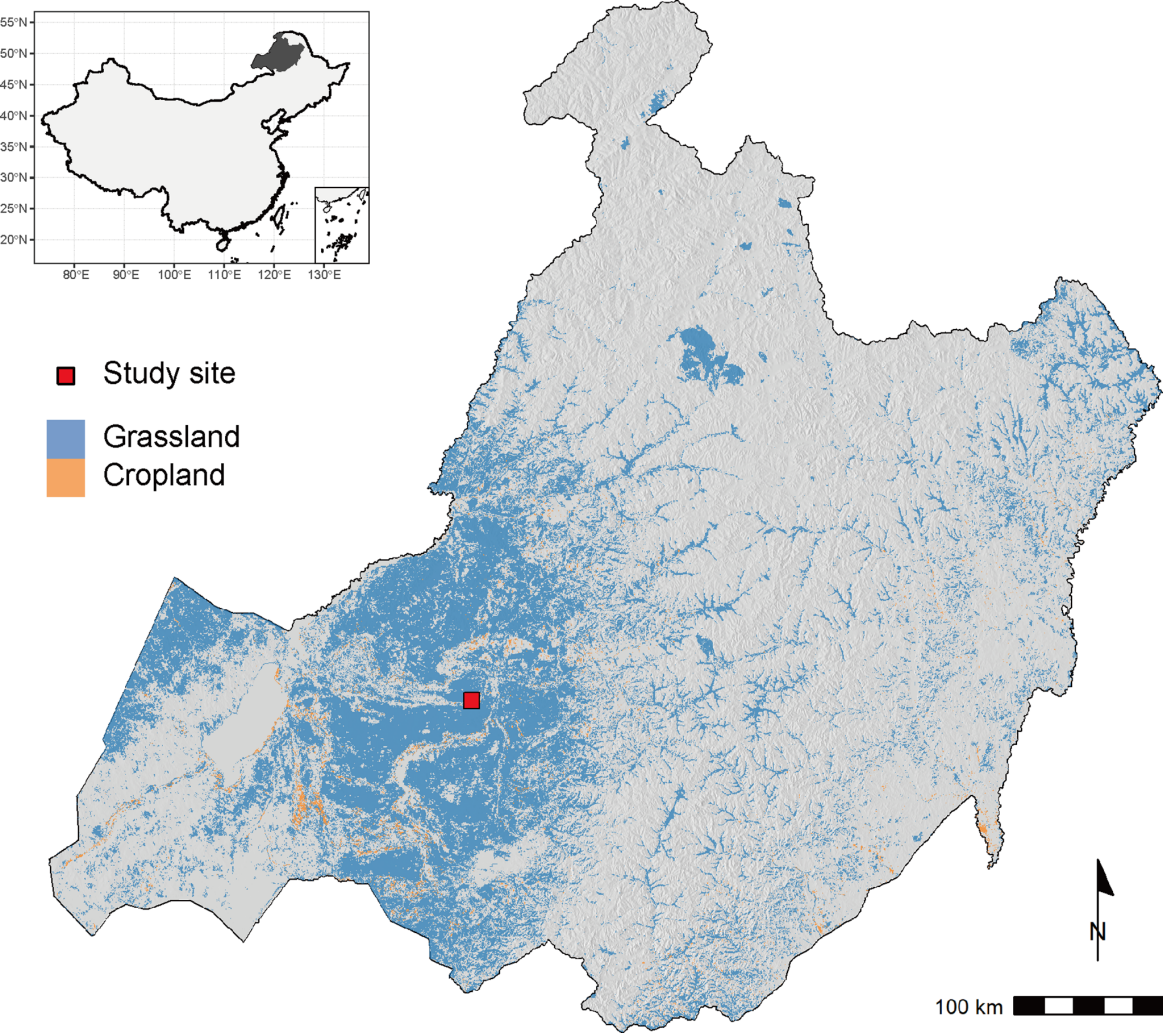
The location of the study site in relation to the regional distribution of grasslands and croplands, which are extracted from ESA CCI land cover v2.0.7 for the year 2015. The underlying terrain is created based on NASA’s SRTM dataset.

**Figure 2:**
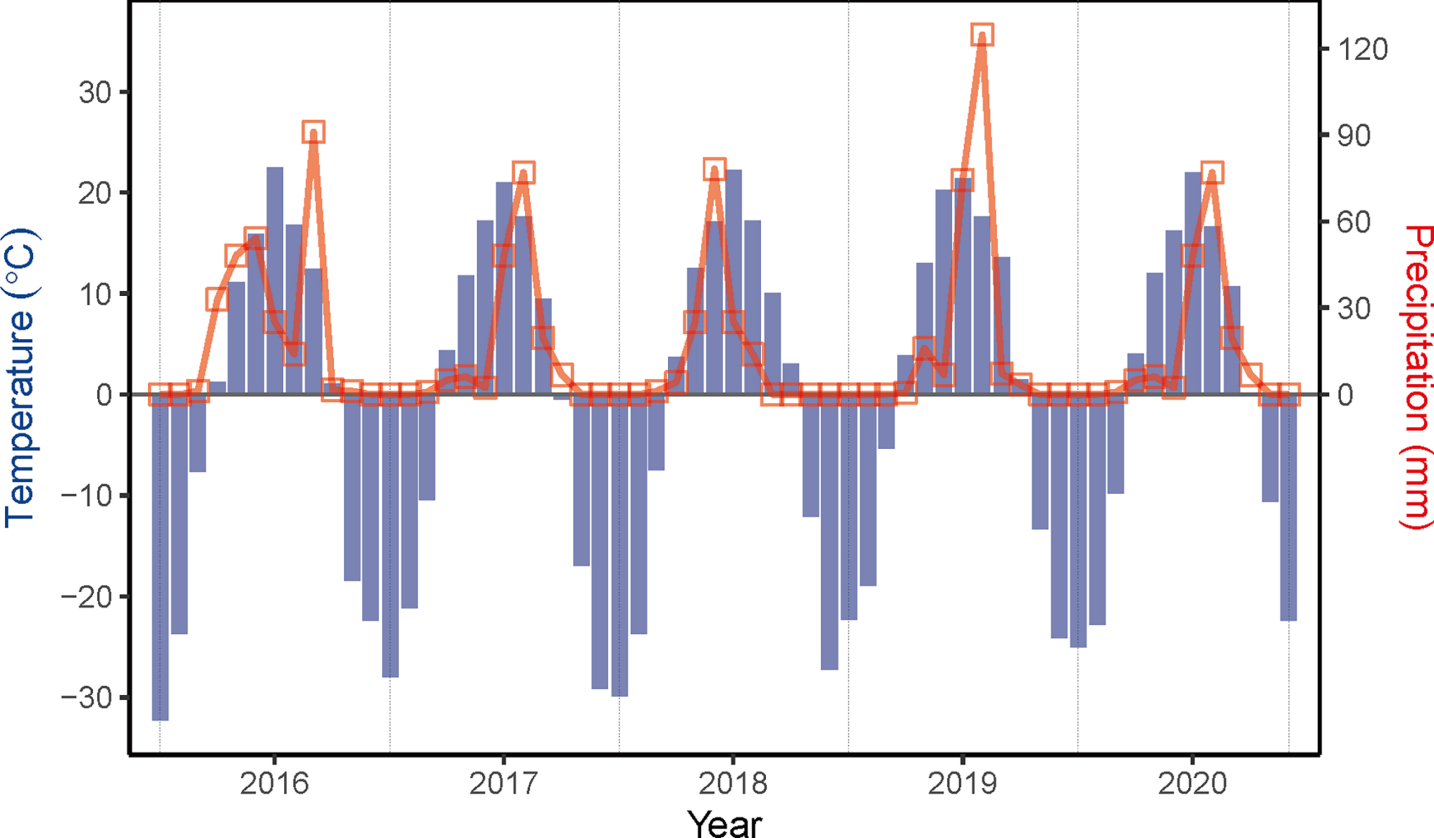
Variations in monthly temperature and precipitation measured on-site from 2016 to 2020.

Field experiments of sown pastures were conducted on a plot of abandoned cropland at the National Hulunber Grassland Ecosystem Observation and Research Station in northeastern Inner Mongolia, China from 2016 to 2020 (**Figure 1**). The previous land use of the abandoned cropland was single cropping of oat (*Avena sativa* L.). A temperate continental climate prevails in the region. The accumulated temperature accounts for 1700-2300 degree-days on the base temperature of 10°C, corresponding to a frost-free period of 85-155 days. The mean annual temperature varied between -2.1 and 0.6°C, as measured on site from 2016 to 2020, while the mean annual precipitation ranged between 148 and 270 mm (**Figure 2**). The dominant soils in the area are classified as Kastanozems (IUSS Working Group WRB, 2015).

### 2.2 Experimental setting and design

**Figure 3:**
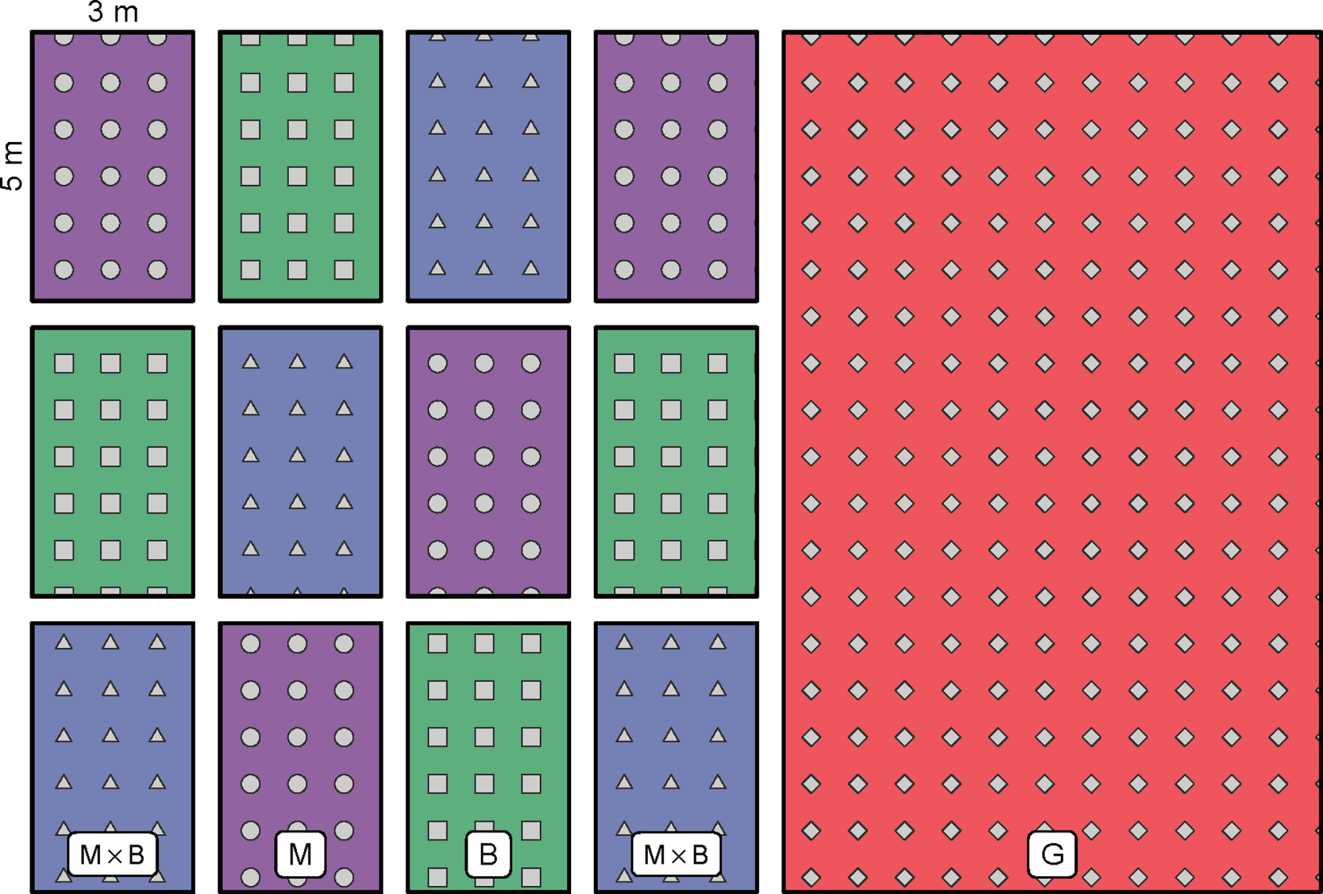
Plot arrangements for alfalfa monoculture (M), smooth bromegrass monoculture (B) and M×B mixture in the field experiment. A large plot of fence-enclosed natural grassland (G) is located next to the grass plantation plots.

A random block design was adopted in the field experiment. Three treatments were included, namely alfalfa (*Medicago sativa* L.) monoculture (M), smooth bromegrass (*Bromus inermis* L.) monoculture (B) and the 1:1 mixture of M and B (M×B). Four replicates were included per treatment. In total, 12 experimental blocks were arranged inside the experimental plot (**Figure 3**). Each block was measured 3 m × 5 m in size. Next to the experimental plot located a larger field of natural grassland (G). A fence was installed in the beginning of 2016 around the whole experimental area to prevent disturbances. Both alfalfa and smooth bromegrass are perennial high-quality forage species that are widely cultivated around the world throughout the dry tropical and temperate regions (Monteros et al., 2014). Alfalfa is a perennial legume which can develop deep taproots; alfalfa’s root nodules have the function of nitrogen fixation (Ilyas et al., 2022). Smooth bromegrass is a perennial grass with fine roots and strong creeping stems, and thus are often used for purposes of soil and water conservation and wind erosion control. Plant seeds were sown manually in rows in June 2016. The inter-row spacing was kept at 30 cm wide. Management was maintained at a minimum level, which only included weed removal and minimal irrigation during extreme drought to keep the plants alive. No other management was exercised. Plant was harvested by mowing two times per year, one in June and one in August. The plot of G is of a typical meadow steppe in the region where its dominant species is *Leymus chinensis*, with a range of associated species including *Stipa baicalensis*, *Carex duriuscula*, *Cleistogenes squarrosa*, *Potentilla bifurca*, *Bupleurum scorzonerifoliu* and some other weeds.

### 2.3 Plant and soil sampling

Plants were sampled twice a year in June and August using a quadrat of 30 cm × 30 cm in size before mowing. Only one sampling was possible for the first year, which took place in August 2016. In each experimental block, the quadrat was placed successively at each of the three randomly preselected localities. In total, 12 samples (i.e., 3 localities by 4 blocks, see **Figure 3**) were taken from each treatment (M, B, or M×B). For G, the same number of samples (i.e., 12 samples) were collected. Plants inside the quadrat were manually cut at 5 cm above soil surface and collected for determination of aboveground biomass in the laboratory. The soil right beneath the quadrat was excavated until 30 cm depth. Plant roots were separated from the soil using a 2 mm mesh sieve, and picked up by hand and washed for determination of root biomass. Both plants and roots were oven-dried in the laboratory at 65°C for 24 hours or until a constant weight was reached. Dry weights of the plants and the roots were measured to derive the aboveground biomass and the belowground biomass, respectively. The oven-dried plant sample was also used to determine plant C and N contents. Plant C content was determined using the potassium dichromate oxidation method (Sparks et al., 1996). Plant N content was determined using a Kjeldahl Azotometer (Type 9840, Hanon Advanced Technology Group Co., Ltd., Jinan) (Sáez-Plaza et al., 2013).

Soil samples were taken in August every year from 2016 to 2020 in the same field mission of plant sampling. A root drill (70 mm inner diameter; 100 mm height, New Landmark Instrument Co., Ltd., Beijing) was used to take soil columns at the depth sections of 0-10, 10-20, 20-40, 40-60 cm, respectively. A subsample was taken out of the soil column at each depth section and kept at the temperature of 4°C for the determination of soil microbial biomass C and N contents in the laboratory using the fumigation-extraction method (Brookes et al., 1985; Vance et al., 1987) after fine roots were carefully removed by hand. The rest of the samples were air-dried for determination of pH, soil organic carbon (SOC) and total nitrogen (TN) contents. Soil pH was measured using a pH probe (type PHSJ-4F, Shanghai Precision Science Instrument Co., Ltd., Shanghai) in the 1:2.5 soil-water solution. SOC was determined using the potassium dichromate oxidation method (Walkley and Black, 1934), while soil TN was measured using the same Kjeldahl Azotometer as for plant N content. Separate soil samples were taken from the field at 0-10 and 10-20 cm depth sections using a 5 cm × 5 cm soil cylinder to determine soil bulk density (BD) using the core method (Petersen and Calvin, 1986).

### 2.4 Data analysis

Multiple comparisons and significance tests were conducted using the analysis of variance (ANOVA) technique coupled with the Tukey Honest Significant Difference method. Trend analysis was conducted by fitting linear regression models between the time variable and the soil variable. Vertical trends were also analyzed. All analyses were processed with the R software, version 4.1.3 (R Core Team, 2022).

## 3 Results

### 3.1 Soil physicochemical properties

**Figure 4:**
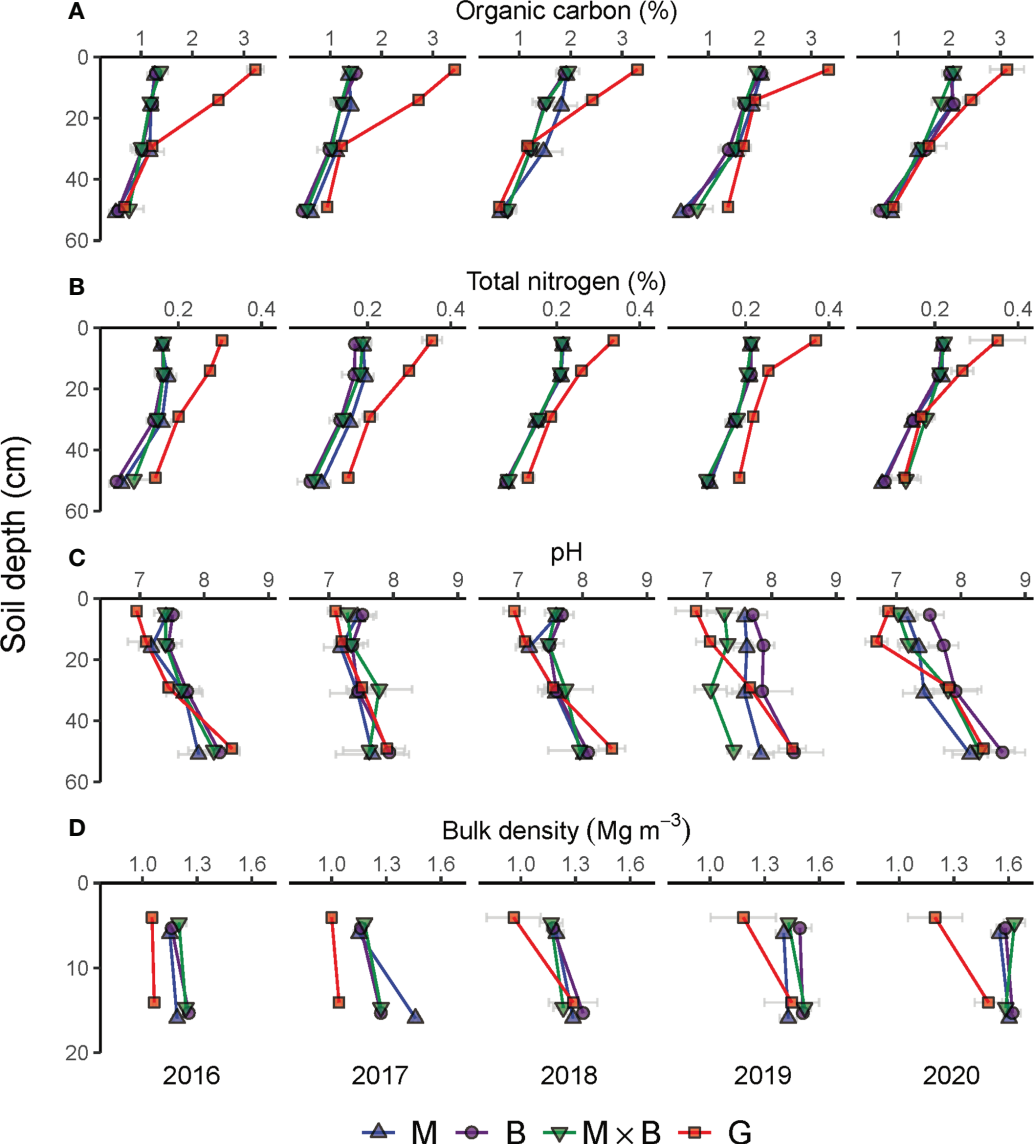
Year-to-year variations in soil physical and chemical properties during the experimental period from 2016 to 2020 under the monocultures of alfalfa (M), smooth bromegrass (B), and the M×B mixture in comparison with a natural grassland (G). Grey horizontal bars represent the range of mean ± one standard error. **(A)**, soil organic carbon content (%); **(B)**, soil total nitrogen content (%); **(C)**, soil pH; **(D)**, soil bulk density (Mg m^-3^).

The year-to-year variations in SOC, TN, pH and BD over the sampling depths of soils under M, B and M×B are presented in **Figure 4**. The physicochemical properties of the soil of G are included for comparison. The evaluation results of the temporal trends of the soil properties are given in **Tables 1** and **2**, while results of vertical trends are given in **Tables 3** and **4**.

**Table 1:** Temporal variations and trends in the physicochemical properties of soils under sown pastures versus the natural grassland from 2016 to 2020.

		Sown pasture				Natural grassland
		*M*	*B*	*M×B*	Mean
Organic carbon (%)						
	SOC_2016_	1.07	1.05	1.09	1.07	1.91
	SOC_2020_	1.60	1.58	1.55	1.58	2.02
	ΔSOC	0.53	0.53	0.47	0.51	0.11
	Trend (Δ yr^-1^)	0.13^**^	0.14^**^	0.13^**^	0.14^****^	0.02
Total nitrogen (%)						
	STN_2016_	0.14	0.14	0.14	0.14	0.23
	STN_2020_	0.16	0.16	0.19	0.17	0.23
	ΔSTN	0.02	0.03	0.04	0.03	0
	Trend (Δ yr^-1^)	0.01	0.01^*^	0.01^*^	0.01^***^	0
pH						
	pH_2016_	7.55	7.73	7.65	7.64	7.48
	pH_2020_	7.52	7.95	7.58	7.68	7.48
	ΔpH	-0.02	-0.22	-0.07	0.04	0
	Trend (Δ yr^-1^)	-0.01	0.08	-0.04	0.02	0
Bulk density (Mg m^-3^)						
	BD_2016_	1.17	1.21	1.22	1.20	1.06
	BD_2020_	1.58	1.60	1.61	1.60	1.34
	ΔBD	0.41	0.40	0.39	0.40	0.29
	Trend (Δ yr^-1^)	0.09^****^	0.11^****^	0.10^****^	0.10^****^	0.09^**^

The slope parameter of a linear model fitted to the year and the soil property is reported as the trend term to represent the rate of change per year. * p < 0.05, ** p < 0.01, *** p < 0.001, **** p < 0.0001.

**Table 2:** Temporal variations and trends in the biological properties of soils under sown pastures versus the natural grassland from 2016 to 2020.

		Sown pasture				Natural grassland
		*M*	*B*	*M×B*	Mean
Microbial biomass carbon (mg kg^-1^)						
	SMBC_2016_	129.27	135.95	191.53	152.25	256.48
	SMBC_2020_	273.64	287.38	247.79	269.60	447.23
	ΔSMBC	144.37	151.43	56.26	117.35	190.75
	Trend (Δ yr^-1^)	45.17^****^	36.82^****^	19.25^**^	33.75^****^	38.14^****^
Microbial biomass nitrogen (mg kg^-1^)						
	SMBN_2016_	24.67	20.05	31.24	25.32	42.85
	SMBN_2020_	63.31	73.25	55.86	64.14	125.98
	ΔSMBN	38.64	53.20	24.62	38.82	83.13
	Trend (Δ yr^-1^)	8.11^***^	12.87^****^	4.23^***^	8.38^****^	15.20^***^
Aboveground biomass (Mg ha^-1^)						
	AGB_2016_	1.72	2.59	2.18	2.17	4.64
	AGB_2020_	7.68	6.78	10.58	8.35	5.04
	ΔAGB	5.96	4.19	8.40	6.18	0.40
	Trend (Δ yr^-1^)	1.53^****^	0.91^***^	2.02^****^	1.49^****^	0.11
Belowground biomass (Mg ha^-1^)						
	BGB_2016_	0.32	0.48	0.40	0.40	5.89
	BGB_2020_	5.85	8.33	8.36	7.51	6.55
	ΔBGB	5.53	7.85	7.96	7.11	0.67
	Trend (Δ yr^-1^)	1.11^****^	1.62^****^	1.86^****^	1.53^****^	0.30

The slope parameter of a linear model fitted to the year and the soil property is reported as the trend term to represent the rate of change per year. ** p < 0.01, *** p < 0.001, **** p < 0.0001.

**Table 3:** Vertical variations and trends in soil physicochemical properties over soil depth in sown pastures versus the natural grassland.

		Sown pasture				Natural grassland
		*M*	*B*	*M×B*	Mean
Organic carbon (%)						
	0-10 cm	1.73	1.76	1.75	1.75	3.28
	40-60 cm	0.63	0.62	0.74	0.67	0.88
	ΔSOC	1.10	1.14	1.01	1.08	2.40
	Trend (Δ cm^-1^)	-0.02^****^	-0.03^****^	-0.02^****^	-0.02^****^	-0.05^****^
Total nitrogen (%)						
	0-10 cm	0.20	0.20	0.20	0.20	0.34
	40-60 cm	0.08	0.08	0.10	0.09	0.15
	ΔSTN	0.12	0.12	0.10	0.11	0.19
	Trend (Δ cm^-1^)	-2.70e-3^****^	2.71e-3^****^	-2.32e-3^****^	-2.58e-3^****^	-4.29e-3^****^
pH						
	0-10 cm	7.44	7.59	7.31	7.45	6.94
	40-60 cm	7.92	8.25	7.89	8.02	8.29
	ΔpH	-0.48	-0.66	-0.57	-0/57	-1.35
	Trend (Δ cm^-1^)	0.01^***^	0.02^****^	0.01^**^	0.01^****^	0.03^****^
Bulk density (Mg m^-3^)						
	0-10 cm	1.29	1.31	1.32	1.31	1.08
	10-20 cm	1.39	1.40	1.37	1.39	1.27
	ΔBD	-0.1	-0.09	-0.05	-0.08	-0.19
	Trend (Δ cm^-1^)	0.01	0.01	4.69e-3	7.84e-3^*^	0.02^*^

The slope parameter of a linear model fitted to soil depth and the soil property is reported as the trend term to represent the rate of change per centimeter of depth. * p < 0.05, ** p < 0.01, *** p < 0.001, **** p < 0.0001.

**Table 4:** Vertical variations and trends in soil biological properties over soil depth in sown pastures versus the natural grassland.

		Sown pasture				Natural grassland
		*M*	*B*	*M×B*	Mean
Microbial biomass carbon (mg kg^-1^)						
	0-30 cm	248.25	253.71	240.37	247.45	378.67
Microbial biomass nitrogen (mg kg^-1^)						
	0-30 cm	47.69	45.07	44.17	45.61	74.94
Belowground biomass (Mg ha^-1^)						
	0-30 cm	4.53	6.49	6.14	5.72	7.17

The slope parameter of a linear model fitted to soil depth and the soil property is reported as the trend term to represent the rate of change per centimeter of depth.

### 3.1.1 SOC and TN

Throughout the study period, SOC at soil depths of 0-60 cm changed consistently across plant species (M, B and M×B). A decreasing trend was observed from the surface to deeper layers (**Figure 4A**). While the SOC content stayed relatively stable for the 20-60 cm layer, the SOC contents at the shallower layers increased steadily from 2016 to 2020 under the influence of plant development. In comparison, the SOC content of the natural grassland soil (G) was found substantially higher than the soil under sown pastures during the entire experimental period (**Table 1**) and across the entire soil sampling depth (**Table 3**). However, it is important to note that the SOC difference between natural grassland and sown pastures was continuously decreasing, from 1.61% in 2016 to 0.75% in 2020, in the 0-20 cm soil layer.

Variations in soil TN content followed a similar vertical pattern that soil TN content decreased with increasing soil depth (**Figure 4B**). However, no sensible temporal patterns were characterized for soil TN at all soil layers.

### 3.1.2 Soil pH

Soil pH showed an increasing trend with soil depth, both for natural grassland and sown pastures (**Figure 4C**). The establishment of sown pastures led to a slight increase in surface soil (0-20 cm) pH compared with that of G. However, a temporal trend in pH variations was not detected (**Table 1**). Differentiations in soil pH among the experimental treatments became obvious in 2019. From 2016 to 2020, the soil pH under B varied between 7.73-7.95, which was slightly higher than that under M (7.52-7.55) or M×B (7.58-7.65). Soil pH under G was largely stable at the level of 7.48 during the entire period (**Table 1**).

### 3.1.3 Soil BD

Soil BD was generally stable in the topsoil (0-20 cm). Nevertheless, soil BD tended to increase with time, especially for sown pastures, as indicated by the positive trend terms in **Table 1**. Sown pasture soils had higher BDs than the soil of G, especially for the top 0-10 cm layer (**Figure 4D**). Differentiations in BD values were hardly observed among M, B and M×B during the entire experimental period. Nevertheless, soil BD tended to increase with time, especially for sown pastures, as indicated by the positive trend terms in **Table 1**. The average BD for soils under M, B and M×B increased from 1.2 Mg m^-3^ in 2016 to 1.6 Mg m^-3^ in 2020 by a margin of 33%.

### 3.2 Soil and plant biological properties

#### 3.2.1 Soil microbial biomass C and N

**Figure 5:**
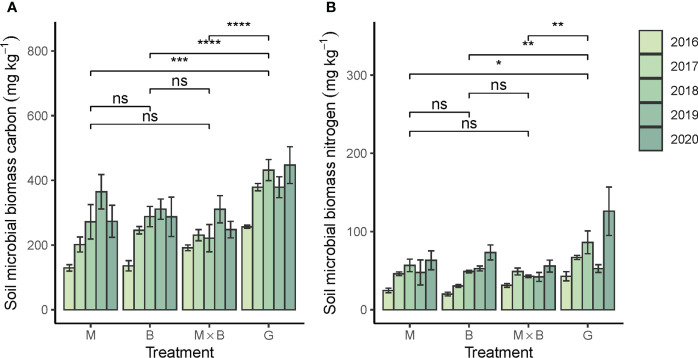
Year-to-year variations in soil microbial biomass carbon **(A)** and nitrogen **(B)** contents under the alfalfa (M) and smooth bromegrass (B) monocultures and the M×B mixture in comparison with a natural grassland (G) during the experimental period from 2016 to 2020. Belowground biomass was measured for 0-30 cm soil depth. Bars and whiskers represent ± σ_M_. * p < 0.05, ** p < 0.01, *** p < 0.001, **** p < 0.0001, ns, p > 0.05.

Both soil microbial biomass C (SMBC) and N (SMBN) were responded positively to sown pastures (**Figure 5**). Significant growth rates in SMBC and SMBN were statistically characterized for soils under all plant species (**Table 2**). The discrepancy in C and N abundance is reflected by the soil microbial C:N ratio (**Table 5**) in comparison to that of the mineral soil or the plant inputs. It is important to note that both SMBC and SMBN abundances under sown pasture were significantly lower than in the natural grassland. No significant difference was observed among the sown pasture plant species (**Figure 5**).

**Table 5:** C:N ratios of the mineral soil, the soil microbial biomass and the plants of alfalfa (M), smooth bromegrass (B), M×B mixture and the natural grassland.

		Sown pasture				Natural grassland
		*M*	*B*	*M×B*	Mean
Soil						
	0-10 cm	8.7	9.0	8.7	8.8	9.6
	10-20 cm	8.3	8.1	7.7	8.0	8.9
	20-40 cm	8.6	8.2	7.8	8.2	7.0
	40-60 cm	7.5	8.0	7.5	7.7	6.0
Soil microbial biomass						
	0-30 cm	5.6	6.2	5.8	5.8	5.5
Plant		14.5	20.5	15.2	16.7	17.9

#### 3.2.2 Plant C and N contents

**Figure 6:**
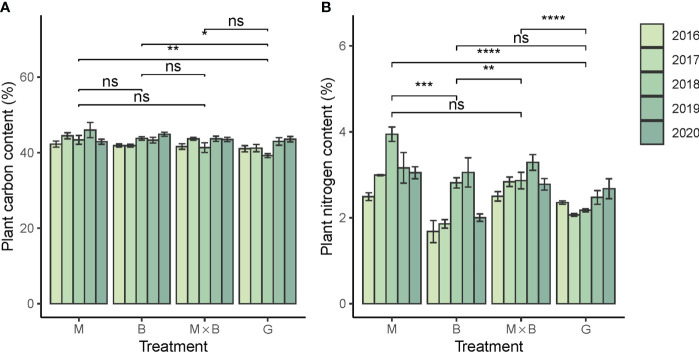
Year-to-year variations in plant carbon **(A)** and nitrogen **(B)** contents of alfalfa (M), smooth bromegrass (B) and the M×B mixture in comparison with a natural grassland (G) during the experimental period from 2016 to 2020. * p < 0.05, ** p < 0.01, *** p < 0.001, **** p < 0.0001, ns p > 0.05.

The plant C contents of alfalfa and smooth bromegrass were measured at comparable levels to each other (**Figure 6A**). However, the N contents showed a significant difference between M and B (**Figure 6B**). The plant C:N ratio of B was higher than that of M, revealing the difference between a grass and a legume (**Table 5**). In comparison, the plant N content of G was significantly lower than that of M, but the difference between G and B was not significant. In terms of temporal variations, plant N content showed an increasing trend especially during the first three years of experiment, while the similar trend was not observed for G. Likewise, no sensible temporal trend was observed for plant C content.

#### 3.2.3 Above- and belowground biomass production

**Figure 7:**
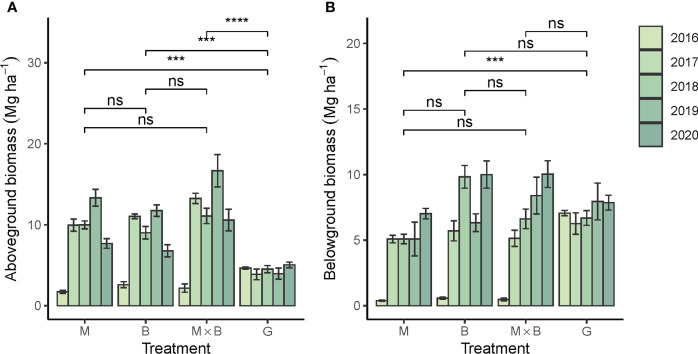
Year-to-year variations in above- and belowground biomass production of the alfalfa (M), smooth bromegrass (B) monocultures and the M×B mixture in comparison with a natural grassland (G) during the experimental period from 2016 to 2020. **(A)**, aboveground biomass production; **(B)**, belowground biomass production in 0-10 cm soil layer. Bars and whiskers represent ± σ_M_. *** *p* < 0.001, **** *p* < 0.0001, ns *p* > 0.05.

The aboveground biomass (AGB) of sown pastures showed a systematic advantage over G, confirming that the productive capacity of M and B were both significantly higher than that of G (**Figure 7A**). Unsurprisingly, the AGB from M×B mixture was observed higher than that of M or B monoculture, although the difference was not statistically significant. The belowground biomass (BGB) of the sown pastures was comparable in magnitude to AGB, showing strong root developments from these forage species (**Figure 7B**). Both AGB and BGB showed a strong temporal trend in plant development at an average rate of about 1.5 Mg ha^-1^ yr^-1^ for sown pastures (**Table 5**). In comparison, the BGB of G varied only slightly during the experimental period at 5.2-6.6 Mg ha^-1^, while its AGB varied at a significantly lower level of 3.9-5.0 Mg ha^-1^.

## 4 Discussion

### 4.1 Soil rehabilitation with sown pastures

**Figure 8:**
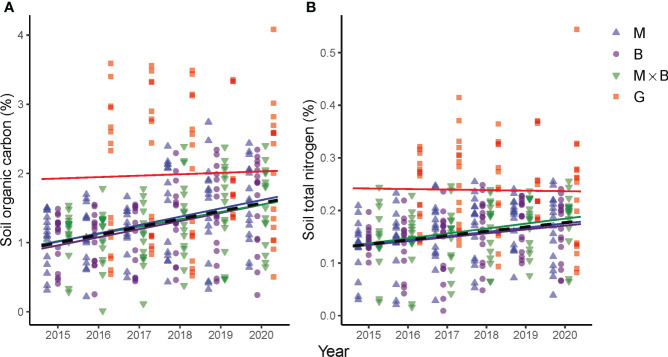
Temporal variations and trends in soil organic carbon **(A)** and soil total nitrogen **(B)** contents at the depth range of 0-60 cm under the alfalfa (M), smooth bromegrass (B) monocultures and the M×B mixture as compared to a natural grassland (G) from 2015 to 2020. The year 2015 is included as a baseline which shows pre-experiment C and N levels. Linear trends are fitted to SOC and TN measurements per treatment, whose color coding is kept in agreement with the legend.

Our results support the general trend in soil quality improvement that was largely established following the reverse conversion of cropland to grassland or the introduction of perennial plant species into the land use system in recent years (Malhi et al., 2002; Jiang et al., 2006). Following the introduction of M and B monocultures and M×B mixture, the soil responded proactively. Two years after the plant establishment, for example, statistically significant increases in SOC and TN had been detected. In the topsoil, the SOC and TN increased by 43 and 41%, respectively, over the baseline levels of 2015. Although the increase in deeper soil layers was slower compared to the topsoil, the average improvements over the entire sampling depth of 0-60 cm were evaluated as 32 and 19%, respectively, for the same period. In addition, substantial improvements were also observed in soil microbial activities, plant root developments and forage yield as an indication of the soil’s productive capacity. Moreover, statistically significant trends were not only characterizable for SOC and TN, but also for biological properties of SMBC, SMBN, BGB and AGB, showing sown pasture’s consistently positive effects on soil conditions. A further scrutinization of the soil’s response in term of SOC and TN as key indicators of soil health (Wiesmeier et al., 2019) in comparison to 2015 (**Figure 8**) – the year preceding the field experiment – revealed that both SOC and TN responded positively to sown pastures, although the magnitude of the response rate in percent change per year was close to each other across the treatments, especially for SOC.

It is worth noting that soil bulk density tended to increase following the conversion from annual cropping to perennial vegetation, which is seemingly contradictory to the common theory that cropland has a higher bulk density than grassland (Wiesmeier et al., 2019). The uniqueness of our observation that bulk density increased following annual cropland conversion to sown pasture is that the degree of soil compaction induced by heavy machinery is much lower than the levels in the USA or Europe (Bauer and Black, 1981; Strebel et al., 2007). The major limitation on the use of heavy machinery in China is that Chinese agriculture is essentially small holder-based (Ye et al., 2016; Ye et al., 2017). The average farm size does not allow heavy machinery. Even in Northeast China, where the size of the dominant state-owned farms is much larger, machinery is mostly light-weighted (Zhang et al., 2006). As such, conversion from a manually tilled arable land to a no-till sown pasture would reasonably cause the bulk density to rise. Similar findings were also reported recently elsewhere in China. Huang et al. (2019) found that bulk density of an alfalfa grassland soil on the Qinghai-Tibet Plateau increased from 1.23 to 1.30 Mg m^-3^ during a six-year period, while Jiang et al. (2007) reported that planted alfalfa caused soil bulk density to rise from 1.15 to 1.21 Mg m^-3^ in three years. On the long run, however, a decrease in soil bulk density under sown pasture to a level comparable to that of the natural grassland would be expectable after the root system becomes matured. Furthermore, no temporal trend was found in soil pH; instead, our data indicated a significant vertical trend, meaning that the deeper soil layers tend to be more alkaline. The explanation for this is that the parent material of the regional soil is alkaline (Hulunber Prefecture Soil Survey Office, 1992).

Both M and B delivered a significantly higher AGB than G. This reflects the performance advantage established through long-term breeding for M and B. The forage production (AGB) was well supported by root developments (BGB). A temporal trend was tested significant not only for AGB and BGB, but also for SMBC and SMBN, showing that the belowground processes were still in rapid development. On the one hand, the obtained BGB results suggested that root development in B was superior to M. This may be an artefact resulted from a limited sampling depth, because the fine roots of B were mostly located in topsoil and well sampled in the 0-30 cm soil depth (Malhi et al., 2002), while the taproot of M was more subsoil oriented and thus less sufficiently sampled (Ilyas et al., 2022). The AGB result of M provided additional support for this artefact hypothesis. Throughout the experimental period, M’s AGB tended to increase at an annual rate of 1.53 Mg ha^-1^, higher than 0.91 Mg ha^-1^ for B. This growth rate of M would not be possible if M’s root development were restricted as shown in **Figure 7B**. Therefore, we call for a deeper sampling depth in practice to better represent the root biomass for taproot plants like M. Nevertheless, the complementarity between the shallow and deep roots of B and M, respectively, can better meet their water requirements, and thus better support AGB accumulation (Yamada, 2014). The importance of our evidence of complementarity was that the effect was limited by the number of interactive species of two, while in other experiments, such effects were characterized based on much higher numbers of species. For example, Furey and Tilman (2021) sown mixed seeds of up to 16 species selected from grasses, legumes and forbs, and observed the complementarity effects on soil fertility only after 23 years.

On the other hand, the associated developments between microbial biomass and the plant biomass provide additional insights into the contributions that the soil microbial communities can make to forage production and at the same time to other soil functions and soil health. As an important labile SOC fraction, SMB plays a fundamental role in controlling soil C and N cycling and aboveground productivity (Li et al., 2019). SMB is also considered an early indicator of changes in soil quality because it may respond rapidly to changes in C supply before such changes can be detected as recalcitrant soil organic matter (Jiang et al., 2006; Yao et al., 2015). We found that in association with the macro-scale responses of the soil under sown pasture in terms of soil C, N and biomass production, soil microbial communities responded proactively too, as indicated by the SMB growth rate which showed no sign of a hindered growth in SMB. This suggests that the growth and turnover in SMB were switched to the root litter, shoot and exudates pathway to obtained energy and nutrient supply (Mooshammer et al., 2014) following the blockade of the aboveground litter pathway (Chen et al., 2021) by forage harvesting. Moreover, previous studies proposed that in addition to input quantity, plant input quality played critical roles in the establishment, development and wellbeing of the soil microbial communities (Cleveland and Liptzin, 2007; Reichel et al., 2018). To this end, we compared the SMB C:N ratio to the plant C:N ratio and the soil C:N ratio, and found that the SMB C:N ratio in this study varied between 5.5 and 6.2, which was well in line with SMB C:N ratios found elsewhere (Cleveland and Liptzin, 2007; Reichel et al., 2018). Variations in plant C:N ratios were fairly narrow, with values centered at 14.5 for M and 20.5 for B, well below the 20-25 range, suggesting that N supply was not limited from the plant inputs (Reichel et al., 2018) and thus the microbial decomposer communities would adaptively decrease the microbial N use efficiency to maintain cellular homeostasis, meaning that excessive N that the soil microbes retained were to be released and subsequently mineralized (Mooshammer et al., 2014). This is in part explained the relatively higher level of TN and lower soil C:N ratios in grassland soils as observed both in sown pastures and in natural grasslands in this study and elsewhere (Jiang et al., 2006). In short, our findings of the statistically characterized temporal trends of SMBC and SMBN, together with the root pathway of energy and nutrient acquisition induced from the plant-microbial-soil C:N ratio linkage, represent direct evidence that the microbial communities were in a steady growth during the first 5 years of sown pasture.

### 4.2 Biomass productivity

Our results showed a systematically higher AGB in sown pastures than in natural grasslands. This seemingly simple observation has great technical significance for natural grassland protection and sustainable livestock production, which in our opinion has not received enough attentions so far. Previous research on grassland degradation in Inner Mongolia has found that livestock rearing in captivity is an effective means to meet increasing market demand of animal products, which can in part displace grazing livestock from and can therefore lessen the production pressure on the natural grasslands (Chen et al., 2017; Robinson et al., 2017). However, livestock rearing in captivity relies on imported forage. The reliability and geographical location of the source of forage import are of great concern. Due to the increasing awareness of environmental protection and ecosystem integrity (Ning et al., 2019), a series of grassland restoration programs, such as the Natural Grassland Restoration Program and the Returning Grazing Land to Grassland Program, have been initiated and implemented. Meanwhile, a range of policy reforms have been instituted since the beginning of the 21st century, which includes the Grassland Eco-compensation Program, the Farmer’s Professional Cooperative Law, and so on. Under these policies and institutions, over half of the croplands in Inner Mongolia should be withdrawn from crop production (Li et al., 2020). In the Hulunber region where this study was conducted, some 150×10^3^ hectares (or 150 kha) of croplands are classified marginal and deemed retirement (Hulunber Land Survey Office, 2021). If these marginal croplands were all utilized by sown pastures, the grazing intensity on Hulunber’s natural grasslands would decrease by a big margin of 38% (**Table 6**). This represents a practical pathway to utilize marginal croplands for sown pastures under the current infrastructures of environmental policies and institutions to achieve the goals of sustainable production, soil rehabilitation and grassland protection.

**Table 6:** The potential effect of sown pastures on the stocking rate of the natural grasslands of Hulunber in 2020.

	Unit	M	B	M×B	Sum
Biomass yield	t DM ha^-1^	4.80	5.25	5.40	–
Actual area	10^3^ ha	20.00	2.00	1.33	23.33
Potential area	10^3^ ha	128.59	12.86	8.55	150.00
Forage production	10^3^ t DM	617.23	67.51	46.18	730.92
Livestock supporting capacity	10^6^ SSU	3.65	0.40	0.27	4.32
Total livestock	10^6^ SSU	–	–	–	11.13
Natural grassland area	10^6^ ha	–	–	–	6.27
Stocking rate	SSU ha^-1^	–	–	–	1.77
Decreased stocking rate	SSU ha^-1^	–	–	–	1.09
Stocking rate difference	%	–	–	–	38.42

DM, dry matter; SSU, stand sheep unit. The livestock supporting capacity of sown pastures is evaluated assuming a 50% usage of marginal croplands in 2020. Sown pasture species: alfalfa monoculture (M), smooth bromegrass monoculture (B) and the M×B mixture. Data source: Hulunber Land Survey Office (2021).

### 4.3 Implications for soil conservation and rehabilitation

The first implication is that soil conservation and rehabilitation need long-term efforts. As our results clearly demonstrated that, no matter which parameters to consider, either soil chemical properties like SOC and TN or soil microbial activities like SMBC and SMBN, the natural grassland outperformed sown pastures during the entire experimental period. Previous research from the Grain-for-Grain Program also showed that stabilization of soil C levels occurred only after 11 and 30 years following conversion to grassland and forest or shrubland, respectively (Deng et al., 2014). More challengingly, soil C decreases were often observed in the first 5 years of reverse conversion. Similarly in a more arid environment of the Loess Plateau, SOC increases in seeded alfalfa grasslands only occurred in the 20th year (Jiang et al., 2006). It is important to indicate that establishment and stabilization of the soil microbial communities require additional time, especially when the plant input is limited in quantity (Chen et al., 2019) or low in quality (Cleveland and Liptzin, 2007). As long-term experiments of alfalfa planation on the Loess Plateau revealed, a stable state in SMB could not be reached in the first 10 years (Jiang et al., 2006). In a biodiversity and soil fertility experiment in the US, Furey and Tilman (2021) spent 23 years before the effect of a mixture of 16 species selected from grasses, legumes and forbs on soil fertility was identified. Recent findings have suggested that soil microbes are likely the workhorse in soil conservation and rehabilitation (Coban et al., 2022), suggests that the establishment of stable microbial communities in the soil, especially when adaptation to a new type of plant input (Cleveland and Liptzin, 2007) or to a new rhizosphere environment (Sokol et al., 2019) is required, probably involves processes that operate at a decadal time scale.

Secondly, sown pasture is a win-win option for building resilience into the food system and at the same time providing multiple ecosystem services. Our results have shown that establishment of sown pastures improved soil quality and raised the soil productive capacity. It is clear that sown pasture-based forage production is itself a valid measure to lessen grazing pressure on and thus to counter degradation of natural grasslands, which is in part contributing to food system resilience (Ye et al., 2010; Robinson et al., 2017). As our previous analyses have found, incorporating perennial forage grasses into agricultural cropping systems or forming mosaic landscapes with the natural grasslands can produce multiple benefits (Xu et al., 2020; Xu et al., 2021). These include, among others, C sequestration, soil fertility improvement, soil and water conservation and wind erosion control. Moreover, a legume-incorporated system has the ability to fix atmospheric N for plant use owing to its symbiotic nature of plant-rhizobium relationship at a rate ranging from 13 to 682 kg N ha^-1^ yr^-1^ (Ledgard and Steele, 1992). This, together with additional benefits of lowering N_2_O emissions from displaced N fertilizers and sequestering C in the soil (Xu et al., 2018), represent a nature-based solution in climate change mitigation.

Thirdly, institutional reforms to facilitate forage grass adoption in wider agropastoral regions are needed by considering harmonization between social development and nature wellbeing, supported by a healthier plant-soil system. Although adopting perennial species into agricultural systems produces multiple benefits, one of the longer-term adverse effects of forage-biomass production, in particular those with taproots such as M, is the possible depletion of deep-layer soil water (Jiang et al., 2006). One solution can be a rotational plantation based on a single life cycle of alfalfa pasture, similar to the rotational grazing system used in grassland conservation (Kassam and Kassam, 2021; Dong et al., 2022). In fact, deep-soil dryness is not unique with forage pastures. Deng et al. (2020) found that soil dryness was extensively associated with vegetation greening across species and space. To avoid irreversible damages that a prolonged soil dryness could make on soil functions at the local to subregional scales, the use of policy tools is not only inevitable but also more effective (Murthy and Bagchi, 2018; Bardgett et al., 2021).

## 5 Conclusions

The following conclusions can be drawn from the results obtained from our five-year field experiment of sown pastures:

- Firstly, our study has clearly demonstrated that sown pasture is an effective soil rehabilitation measure. Soil’s responses to sown pastures are consistently positive across the soil physicochemical and biological properties, although the response rate of the (micro)biological properties are relatively more proactive.
- Secondly, sown pastures have systematic advantages over the natural grasslands in biomass production. This means that if the available marginal cropland resources are properly used for sown pastures, the grazing pressure on the natural grasslands can be substantially lowered through the pathway of livestock rearing in captivity. Our analysis shows that a 30% decrease in the grazing intensity in the Hulunber region is foreseeable if all marginal croplands are converted to sown pasture in this traditionally pastoral region.
- Thirdly, despite these improvements, the major quality indicators of the sown pasture soil are not comparable to those of the natural grassland soil in the short run. Our analysis suggests that the processes involved in soil rehabilitation and restoration are likely operating at the decadal time scale.
- Last but not least, we hereby advocate the adoption of a long-term mindset in soil conservation and rehabilitation and call for policy adjustments to facilitate the incorporation of sown pastures into the current plant-soil systems especially in the semiarid to arid agropastoral regions in northern China.

## Data availability statement

The original contributions presented in the study are included in the article/supplementary material. Further inquiries can be directed to the corresponding author.

## Author contributions

All authors listed have made a substantial, direct, and intellectual contribution to the work and approved it for publication.

## Funding

This work was supported by the National Natural Science Foundation of China (grant number: 41703081), and the China Agriculture Research System (grant number: CARS-34).

## Conflict of interest

The authors declare that the research was conducted in the absence of any commercial or financial relationships that could be construed as a potential conflict of interest.

The Reviewer HW declared a shared affiliation with the author LX, YN, WX at the time of the review.

## Publisher’s note

All claims expressed in this article are solely those of the authors and do not necessarily represent those of their affiliated organizations, or those of the publisher, the editors and the reviewers. Any product that may be evaluated in this article, or claim that may be made by its manufacturer, is not guaranteed or endorsed by the publisher.
